# Trauma-informed care in the emergency department: concepts and recommendations for integrating practices into emergency medicine

**DOI:** 10.1080/10872981.2023.2178366

**Published:** 2023-02-17

**Authors:** Audria Greenwald, Amber Kelly, Listy Thomas

**Affiliations:** a Department of Medical Sciences, Frank H. Netter School of Medicine at Quinnipiac University, North Haven, CT, USA; b Department of Social Work, Quinnipiac University School for Health Sciences, North Haven, CT, USA

**Keywords:** Trauma-informed care, emergency medicine, psychological trauma, medical trauma, patient-centered care

## Abstract

The experience of psychological trauma is common and has become even more prevalent during the COVID-19 pandemic for both health care workers and the general population [1–3]. Traumatic experiences can have varied and lasting physical and mental health effects on patients, beyond what we are privy to in the acute environment of the emergency department. The effects of these prior traumatic experiences can be exacerbated by interaction with the healthcare system, and yet emergency medicine physicians have no standardized methods for working with patients in a trauma-informed way. The systematic implementation of trauma-informed care (TIC) practice requires the cooperation of multiple domains within the health care system, including focus on the physical environment, direct care, and administrative practices. Here we provide recommendations specific to emergency medicine for the development and implementation of TIC in the regular patient-clinician interaction, situated within the context of the TIC framework as outlined by the Substance Abuse and Mental Health Services Administration (SAMHSA) [4].

## Introduction

In the United States and worldwide, exposure to one or more traumatic events is a highly prevalent experience among the general population and is associated with both psychological and physical sequelae [6,7]. Based on a survey study of 24 nations, the global prevalence of exposure to one or more traumatic experiences is estimated to be 70.4%, with the majority of those having multiple traumatic exposures [6]. Prevalence of a history of traumatic experiences and the type of experience varies across and within populations, with increased prevalence among Black, Indigenous, and other communities of color as well as those in poor and urban areas [5,8,9]. It is estimated that two-thirds of all individuals have experienced at least one traumatic event before the age of 18 [7].

Experiences of traumatic events can have varied and lasting physical and mental health effects on patients, which can be further exacerbated by interaction with the health care system [7,10–13]. Medical events in themselves can be a cause of traumatic stress, both for children and adults [14–17]. One study found that 10–20% of adult physical trauma patients admitted for care developed PTSD post-incident and an additional 14–28% went on to develop acute stress disorder (ASD) [14]. Another study found that admitted injured pediatric trauma patients showed rates between 10–69% for PTSD and 14–28% for ASD [18].

With this prevalence of trauma, we can recognize that many of the patients we meet in the emergency department (ED) have been impacted by traumatic events at some point in their lifetime and it is likely that many of these patients continue to experience negative impacts of these past events. Even before the COVID-19 pandemic, individuals with a more substantial history of psychological or physical trauma utilized emergency care at higher rates than the general population [19–21]. We must also acknowledge that in the case of the acute traumas we care for every day in our clinical encounters the trauma experienced by the patient does not end when they are dispositioned out of the ED. As such, it is imperative that emergency medicine recognizes the growing body of literature on the benefits of trauma-informed care (TIC) to better understand the context in which a patient presents to the ED, methods to provide TIC, and how to mitigate retraumatization.

There have been longstanding calls to bring a trauma-informed approach to other aspects of medical care, and it is time we hear and respond to this call for emergency medicine [9,22]. As we recognize the prevalence of exposure to traumatic events in our current society, we recommend that TIC be instituted as universal precautions in all emergency care, just as we consider hand hygiene and appropriate personal protective equipment [23].

## Defining trauma

The Substance Abuse and Mental Health Services Administration (SAMHSA) defines individual trauma in the context of the ‘Three E’s’ as ‘the result of an **event**, series of events, or set of circumstances that is **experienced** by an individual as physically or emotionally harmful or life threatening and that has lasting adverse **effects** on the individual’s functioning and mental, physical, social, emotional, or spiritual well-being.’ [5] A traumatic exposure may be experienced directly, witnessed by a loved one, or can be vicarious as in those witnessed by first responders responding to traumatic events [24].

## Types of trauma

Traumatic exposures encompass a diverse group of experiences at the individual, interpersonal, collective/community, and historical level. Traumatic events can include both impersonal trauma (ecological events such as hurricanes, tsunamis, and landslides), or interpersonal traumas (events between people, such as child neglect and abuse, intimate partner violence, sexual assault, and human trafficking) [25]. We know that those who experience interpersonal violence, especially those who experience violence over time (rather than a one-time acute event), tend to show worse long-term outcomes [25–27]. The literature also shows that for survivors of interpersonal violence, the closer the person who inflicts the violence is to the survivor, the more impactful the event (for instance, stranger assault vs. assault by a family member) [25,27]. We treat survivors of both types of traumatic events in emergency medicine, whether in the acute aftermath or years later in related or unrelated health or mental health emergencies. Community violence involves the ‘exposure to intentional acts of interpersonal violence committed in public areas by individuals who are not immediately related to the victim’ and include gang violence, public shootings, war, and terrorist attacks [28]. Historical trauma refers to the ‘complex and collective trauma that is experienced over time and across generations by a group of people who share an identity, affiliation, or circumstance’ [9]. Historical trauma pertains to populations whose relatives and ancestors were impacted by mass traumatic events, generally violent in nature, such as enslavement in the US, the Holocaust, Japanese internment camps, and racism and racist policies towards Black, Indigenous, and other People of Color [9].

## Impact of trauma on health, disease, and neurobiology

The 1998 Adverse Childhood Experiences (ACE) study elucidated the ways in which a history of traumatic experiences during childhood predisposes people to the development of chronic physical and mental health conditions. The study utilized a ten-question ‘yes’/‘no’ questionnaire which sought to quantify and identify the traumatic experiences participants were exposed to in childhood. The study found that approximately two-thirds of respondents reported one ACE, and that the likelihood of additional exposure increased by 87% if the person had one exposure of any type. 16.67% of respondents reported 4 or more ACEs. The study also revealed a positive correlation between the number of ACEs a person has with poor health outcomes, including higher rates of depression, suicide attempts, substance use including cigarette smoking, liver disease, heart disease, and chronic obstructive pulmonary disease [7].

The physiological response to chronic stress and traumatic exposures is complex and is dependent on a variety of factors including age, genetics, history, and available support and resources [5]. Dysregulation of the hypothalamic-pituitary-adrenal (HPA) axis inhibits the return from a stressed/activated state to a homeostatic baseline and may occur due to chronic activation of the HPA axis [29–31]. This chronic exposure to endocrine and neural responses to stress is related to the development of chronic health problems such as those identified in the ACEs study [7,31,32]. It is critical that emergency health providers understand the prevalence of experience of traumatic events, as well as the potential long term negative healthcare impacts.

## Medical trauma and re-traumatization in healthcare

There is often increased utilization of emergency and same-day services by those with a more significant history of trauma and decreased utilization of primary care and mental health services [19–21]. However, it is important for clinicians to remember that interaction with the health care system can be a highly stressful and potentially triggering experience for survivors of trauma, and these experiences may lead to further medical trauma and/or re-traumatization. Medical trauma pertains to the psychological and physiological response of patients and their families to pain, injury, serious illness, medical procedures, and invasive or frightening treatment experiences [28]. Re-traumatization can occur due to a previous history of trauma in healthcare, fear and confusion, lack of privacy, stress associated with undergoing procedures, physical touch and removal of clothing, vulnerable physical positions, triggering during interviews, and financial stress [33].

## Defining trauma-informed care

Trauma-informed care (TIC) is defined by SAMHSA as ‘an organizational structure and treatment framework that involves understanding, recognizing, and responding to the effects of all types of trauma’ [5]. The framework of TIC emphasizes physical, psychological and emotional safety for patients and providers, and it helps survivors rebuild a sense of control and empowerment. These principles can be applied universally to all clinical interactions, and trauma-informed training and policies have been implemented in other health care fields as well as in medical education [13,34–39].

The development and implementation of a TIC model requires the acknowledgment of the ‘4 R’s’ as defined by SAMHSA:

- **Realization** of the widespread impact of trauma and understanding of potential paths for recovery
- **Recognition** of the signs and symptoms of trauma in patients, families, staff, and others involved in the system
- **Response** by fully integrating knowledge about trauma into policies, procedures, and practices
- Active **resistance** against re-traumatization [5]

Furthermore, SAMHSA advises that the development of a TIC model requires sensitivity and prioritization toward the following key principles [5]:

- **Safety**
- **Trustworthiness and transparency**
- **Peer support**
- **Collaboration and mutuality**
- **Empowerment of voice and choice**
- **Cultural, historical, and gender issues**

SAMSHA’s principles assume an underlying goal of TIC is to create an environment of physical and psychological **safety**. In the ED, this means access to healthcare with mitigation of potentially retraumatizing experiences. **Trust and transparency** should be maintained throughout the system, with patients able to understand what is happening at each stage of the encounter and why. **Peer support** should be utilized among providers and patients alike, allowing for an environment of supportive engagement throughout the ED. Patient care should utilize practices of interprofessional team-based care and patient-centered **collaboration and mutuality** that **empower** the patient to participate in the plan of care, with specific focus on patient **voice** and **choice** within that plan. Lastly, a TIC approach also recognizes the intergenerational and systemic violence and traumatization that continues to occur within our institutions, with specific focus on **cultural**, **historical**, and **gender-based violence** [40–43].

The systemic implementation of TIC requires the cooperation of multiple domains within the health care system, including focus on the physical environment, direct care, and administrative practices. A 2022 systematic review evaluated the current data regarding TIC interventions specifically in the emergency department and found that educational interventions, collaboration between patients, health professionals, and community resources, and patient and clinician safety were the prevailing themes [44]. Here we seek to expand upon existing literature with detailed recommendations for TIC interventions in the emergency department setting.

## Methods

The general background for defining trauma and trauma-informed care is based on SAMHSA’s structure and definitions as they are a leading entity in the field [5]. The recommendations provided here are based on results from SCOPUS and PubMed searches performed using search terms including ‘trauma-informed care’, ‘trauma-informed care AND emergency medicine’, and ‘trauma-informed care AND acute care’. A narrative review approach was utilized. Articles which pertained only to physical trauma or were markedly outside of the scope of emergency medicine, such as long-term outpatient management, were excluded. While some of these recommendations may be thought of as general best practices in patient care or fastidious, we include them here with the intention that they be considered in the context of treating patients with history of traumatic exposures.

## Trauma-informed care in the emergency department: practice recommendations

TIC acknowledges pertinent patient history related to past or current traumatization in assessment and plan of care, while trauma-denied care ignores such pertinent history. Trauma-informed practices should be considered universal precautions and can be modified to work within the unique environment of the ED. In the individual physician-patient encounter, TIC skills can be implemented using the following strategies, linked to the specific principles as outlined by SAMHSA: Safety, trustworthiness and transparency, collaboration and mutuality, empowerment of voice and choice, and consideration of cultural, historical, and gender issues. These recommendations are described in Tables 1–4.

**Table 1.** Starting the encounter: recommendations for introducing trauma-informed practices into the beginning of the encounter and creating a psychologically safe environment.

Recommendation	Rationale
Clarify the patient’s name, pronunciation, and pronouns	Some names are difficult to pronounce, but by taking the moment to say a person’s name correctly, the patient is made to feel as though the provider does care about them and sees them as an individual. Routinely clarifying a patient’s name and pronouns and sharing this information with the care team will reduce misnaming or misgendering a patient. When a mistake is made in name or pronoun, it should be repaired sincerely with an honest apology, rather than ignored or rationalized [45,46].
Prioritize safety and privacy by addressing others in the room	When a patient presents with others confirm that the patient wants others with them. The onus should not be on the patient to request that the other person(s) leave the exam, and should be addressed by the provider, to protect the patient [34].
Position yourself at the same level with the patient	Minimize the physical representation of the patient-provider status/power differential by being at the patient’s level. This can be achieved by either sitting at or below the patient, or by raising the exam table or gurney such that the patient is at the provider’s height.
Be aware of negative reactions to patients	Take a moment to be mindful of and address your own internal experiences, anxieties, and wish to control the outcome with patients. Slow down. Be aware of potential compassion fatigue, victim blaming, dismissiveness, savior complexes, taking on your patient’s battles, etc [47]. As people react to their own traumas, they can sometimes show symptoms that are difficult for healthcare providers to understand or navigate. This can include combativeness, destructive health behaviors (drugs, risk taking, etc), or other behaviors that are difficult to understand. Adaptations and coping mechanisms can lead to symptoms that make others anxious, uncomfortable, or frustrated. A history of complex trauma can also create an interpersonal style that may be difficult for both patients and clinicians. Healthcare providers can at times find themselves avoiding certain patients, dismissive of their needs, or outright angry at the choices patients are making in their personal lives. The more providers can be aware of such reactions, the less likely they are to unconsciously make decisions that may lead to inequitable healthcare practices or negative healthcare outcomes for patients.
Be mindful of choices made in the management of agitation	Agitation is a common and major concern in the ED, and its occurrence and management can be dangerous for both the patients and hospital personnel. Approaches to de-escalation should be thoughtful of both the safety of the patient and provider, as well as consider the ways in which management may be traumatic, such as with premature involvement of security personnel and physical restraint. Agitation may be a result of trauma, and this possibility should be taken in account during de-escalation. There are many published guidelines which provide methods for decreasing trauma in de-escalation. Additionally, if resources provide, allowing for decompression in spaces designed as such, may be particularly useful in patients with autism spectrum disorder and mental illnesses [48]. If recourses are not available, then minimizing personnel in the room and decreasing other stimuli may be useful [48,49].

**Table 2.** Patient interview: recommendations for implementation of trauma-informed practices in the patient interview.

Recommendation	Rationale
Use language interpreters when necessary	Patients who are non-English speakers, who have limited English proficiency, or use sign language must be offered professional medical interpreting services. While many patients present with an English-speaking family member or friend, there is risk of incomplete, incorrect, or omitted translation for both accidental and purposeful reasons. Use an interpreter when the provider is not fluent in the patient’s language. This assures that the patient receives all the information they need and can ask questions as desired.
Avoid language that stigmatizes and/or places blame	Development of a safe environment includes minimizing the use of stigmatizing language, including: “frequent flier”, “addict”, victim”, “non-compliant”, “difficult”, “entitled”, “drug-seeking”, etc. To develop psychological safety in your encounters, change the narrative of the history taking from “What’s wrong with you?” to “what happened to you?” These changes improve respect towards the patient and imply that the situation they are in is not an aspect of their character, but of the things they are experiencing or have overcome [54].
Prepare the patient for what to expect and allow time for questions	Preparing the patient for what they can expect, both for the duration of the visit and for any procedure allows the patient to prepare themselves for what they will experience. Questions should be encouraged during this conversation. Consider asking “What questions or concerns do you have?” or “What part of the exam are you most concerned about?”

**Table 3.** Physical exam and procedures: recommendations for a trauma-informed physical exam.

Recommendation	Rationale
Mitigate vulnerability and increase patient comfort with clothing and draping	Being in the ED is a stressful and vulnerable experience which is aggravated by the common necessity to disrobe. One way to improve patient comfort and to avoid triggering survivors of sexual violence is to allow the patient to keep as much clothing as possible, while still maintaining the capacity for an effective exam. When a patient must be disrobed, maintaining patient modesty improves comfort. When a robe or drape must be moved, requesting that the patient move the article themselves grants them more control over what is happening to them [39].
Guide the patient through the exam with supportive language	When performing any physical exam or procedure, describe what will be happening and why, and give warning before touching the patient. Encourage the patient to tell you if they are uncomfortable, in pain, or afraid prior to initiating the exam. In cases when the patient is hearing or visually impaired, take care to inform the patient of your presence so as not to startle, and before each physical interaction. Allowing the patient some control over the pace of the procedure or exam, when possible, allows them to maintain some control over what is happening to them and can decrease the feeling of being trapped. Another way in which language can be modified is through use of de-personalized terms- omitting “your” and replacing it with “the” and omitting the phrase “for me” while doing portions of the physical exam, such as in “take a deep breath for me”. Do not request that they “relax”, instead use a phase such as “open” or “release this muscle”, when necessary. Providing clear, consistent information with appropriate boundaries which actively seeks to avoid terms which may be linked to traumatic experiences, or which contain unintentional innuendo should be avoided [39,55].
Defer Exams and Procedures that may not need to be done	Sometimes, the harm of the exam via mistrust and re-traumatization may outweigh the benefits, such as the experience of a pelvic exam for persons with a history of sexual assault.
Position yourself within the patient’s line of sight	When performing a physical exam or doing a procedure, remain in the patient’s line of sight and if possible, at the same eye level as much as possible. Examples include performing a pulmonary exam from the patient’s side, a thyroid exam from the front, and positioning the patient during a pelvic exam such that they are propped up in a way that they can see you [39].
Be Clear in Describing Procedures	When a procedure or sensitive physical exam must be performed, consider modifications to improve patient comfort. For example, a patient may be taught to self-insert the speculum during a pelvic exam.

**Table 4.** Closing the visit: recommendations for using trauma-informed practices to close the visit and prepare the patient for admission or disposition.

Recommendation	Rationale
Utilize shared decision making	By empowering the patient to take part in the decisions surrounding their care, the patient is less likely to feel trapped and outside of the locus of control. Several frameworks for shared decision making exist, but all models center around the deliberate consideration of advantages and disadvantages of evidenced-based options as well as the patient’s preferences and goals of care [50].
Assure appropriate resources are shared with the patient	Provide appropriate referral information to local, community, and national resources.
Utilize the teach-back method	In stressful and traumatic situations, especially when a person has a significant history of trauma or post-traumatic stress disorder, dissociation or altered or impaired memory formation can occur [51,52]. When closing a patient visit, it is beneficial to utilize strategies to help confirm patient understanding, such as the teach-back method, which has been shown to improve patient retention [53]. The teach-back method asks the patient to use their own words to tell you their follow-up and discharge instructions.
Encourage questions	Patients may have questions that they do not feel comfortable bringing up due to multiple reasons. Ask questions like “What questions do you have for me?” instead of “Any questions?” to create space for questions and make it clear that they are welcomed and expected.

## Universal screening

The emergency department, as a critical point of access to care, is often seen as an opportunity to screen patients who may otherwise be less likely to have recommended screenings for individual and population health measures due to poor access to primary care [56]. In order for screening in the emergency department to be beneficial and efficient, several factors must be considered, including but not limited to minimizing burden on the department and health care system, appropriate follow-up for results, adequate resources for addressing positive screens, and overall patient benefit [56]. Routine and universal screening for traumatic experiences should be performed if the personnel and resources are available to appropriately manage a positive screen [56].

However, even if formal universal screening is not to be performed, the provider can ask the patient if there is a portion of the encounter or exam they are most concerned about and can address those concerns informally. These queries may be accompanied by phrasing which makes it clear that these are questions asked of all patients [39].

Some language for screening could include:

*‘Have you ever experienced trauma in your previous medical or other life encounters?’* or *‘Have you experienced anything that makes seeing a doctor difficult or scary for you?’* [57]

## Peer support – trauma-informed workplace

Trauma-informed care is also critical in fostering a workplace which minimizes the trauma of those who work there. People who work in the helping professions are more likely to have experienced both personal and workplace trauma than the general population [58–61]. Additionally, by the nature of the work in the ED, clinicians, nurses, and all others who work in the environment are susceptible to workplace trauma, vicarious trauma, and re-traumatization of their own histories of traumatic experiences. This susceptibility to vicarious trauma is further increased by the chronic work stress, burnout, and emotional fatigue that is experienced by clinicians. Furthermore, in such emotional states, clinicians are less able to provide care which is empathetic and sensitive to the psychological needs of the patient, and as such, quality of care declines and there is increased risk of error [9]. This is likely to be exacerbated by the COVID-19 pandemic, as supported by studies which report increased anxiety, posttraumatic stress disorder, and other mental health disorders in health care workers on the front lines of the pandemic [1,52,4,62,63]. To improve the capacity of clinicians to provide TIC, attention to their own psychological wellb**e**ing is also of utmost importance. Workplaces can become trauma-informed by participating in increased knowledge sharing and training in TIC, instituting practices such as appropriate paid time off and leave policies, implementing peer support groups to facilitate peer discussion to prevent secondary traumatization, and providing reasonable spaces for rest.

## Challenges and solutions

While some aspects of TIC can be more readily implemented in the ED, there are challenges which may arise due to the limitations of this environment. Notable challenges include the potential urgency of a presentation, limited duration of the encounter, and the single-visit nature of the ED. The treatment of the psychological effects of trauma often requires multiple appointments over extended periods of time. As such, the treatment of trauma-associated mental health disorders and the processing of trauma is often addressed in a longitudinal relationship with a behavioral health provider or in the setting of other long-term relationships, such as with a primary care provider. While this type of relationship and treatment is not applicable in most ED visits, there is the opportunity for the emergency clinician to utilize TIC practices as outlined above to mitigate the possibility of retraumatization, and to minimize medical trauma experienced while the patient is in the clinician’s care. It is also an opportunity to provide referrals to services through which the patient may develop long-term relationships to address their trauma.

## Summary: trauma-informed care as universal precautions

Exposure to traumatic experiences, with or without the development of lasting physical and mental health sequelae, is extremely common in the general population, and even more so in the population with chronic physical and mental health conditions and substance use disorders. Trauma affects all of us- patients, clinicians, and our communities. The utilization of TIC practices in the ED is critical to mitigate active traumatic exposures and to prevent traumatization and retraumatization during interaction with the health care system. While the urgency of some encounters in the ED presents challenges to the practice of TIC at all points of care, the trauma-informed framework should be utilized throughout the encounter. By applying TIC practices as universal precautions- applying them to all patient encounters, regardless of knowledge of a patient’s trauma history, we can prevent re-traumatization and protect survivors.
