# Correction

**DOI:** 10.1080/10872981.2023.2206681

**Published:** 2023-04-27

**Article title**: Trauma-informed care in the Emergency Department: Concepts and recommendations for integrating practices into emergency medicine

**Authors**: Audria Greenwald, Amber Kelly, Listy Thomas

**Journal**: *Medical Education Online*

**Bibliometrics**: Volume 28, Number 01, pages 1-8

**DOI**: https://doi.org/10.1080/10872981.2023.2178366

It has been noted by the corresponding author that the author, Tina Mathew, was accidentally omitted from the author list during submission of the manuscript. She was involved in this project and in its revision. An updated author list has been placed below:

Audria Greenwald^a^, Amber Kelly^b^, Tina Mathew^c^ and Listy Thomas^a^

^a^Department of Medical Sciences, Frank H. Netter School of Medicine at Quinnipiac University, North Haven, CT, USA; ^b^Department of Social Work, Quinnipiac University School for Health Sciences, North Haven, CT, USA; ^c^Department of Emergency Medicine, Weill Cornell Medicine New York, NY, USA

This correction has not changed the description, interpretation, or the original conclusions of the article. The authors apologize for any inconvenience caused.

